# 

**Published:** 1895-07

**Authors:** 


### Literary Note.

HEALTH, SANITATION AND CLIMATOLOGY OF THE SOUTHERN STATES is the name of a quarterly journal that appeared in the beginning of this year. It is edited and published at Washington, D. C., by Dr. Walter C. Murphy. It is a fine specimen of journalism, both as regards its quality and mechanical execution. The April issue contains an illustrated article on the topography, climate and resources of North Carolina, and is written by Hon. Elias Carr, governor. We hope this magazine will attain the success that it so richly deserves.

### Miscellany.

### INDEX TO VOLUME XXXIV.

PAGE.

AARON, CHARLES D., M. D., Antitoxin in Diphtheria . . . 656
Academy of Medicine Notes . . 46
Address to Graduates, Niagara University Medical College . . . . . 1
Ambulance, Electric Railway . . . 426
American Medical Colleges, Association of . . . . . . 63
Medical College Association . . . . . . . . . 754
Andrews, Judson Boardman, M. D., Memorial . . . . . . . 93
Dr. J. B. and the Buffalo State Hospital . . . . . 118
Anemia, Pernicious . . . . . . . . 713
Angell, Edward B., M. D., Observations on an Anencephalic Monster 462
Antitoxin, Successful use of in Diphtheria . . . . . . . . 117
Treatment of Diphtheria with . . . . . . . . . 400
in Malignant Diphtheria . . . . . . . . . 473
Appendicitis . . . . . . . . 223
Hydrotherapy in . . . . . 282
Argon, discovery of . . . . . . . 557
Association of American Medical Editors . . . . . . . . . . . . 755
Astigmatism, Operation to Correct 102

BACTERIOLOGICAL Bureau, Department of Health . . . . 596
Bacteriology, a Course in . . . . . 255
Bartlett, F. W., M. D., Enteroclysis in Enterocolitis and Zymotics 663
Baths, Free Public . . . . . . . 618
Benedict, A. L., M. D., Atonic Dyspepsia . . . . . . . . . 334
Metric Prescriptions . . . 160
Bissell, William G., M. D., work in Bacteriological Bureau . . . . 596
Board of Health Notes . . . . . . 502
Books received . . . . 59, 126, 183, 256
315, 381, 444, 510, 573, 636, 703, 767
Breast, Cancer of . . . . . . . . . 397
Buffalo Medical Journal, two new members of staff . . . . . . . . 562
Bust, a Neurological . . . . . . . 470

PAGE.

CALCULI, Vesicle in the Female . . . . . . . . . 536
Car, new Railway Hospital . . . . 562
Carnot, President, Death Wound of 37
Cataract, Immediate Capsulotomy following Removal of . . 101
Morphine to prevent Prolapse of Iris in Extraction of . . . . . . . . 103
Celiotomy versus Laparatomy . . . 424
Chancre, Treatment of . . . . . . 110
Charity Organization Society of Buffalo . . . . . . . . . . . . . 365
Children, Dosing of . . . . . . . 614
Christian Science and Faith Cure in New Hampshire . . . . . . . . 685
Cinnamon, Essence of as an Antiseptic . . . . . . . . . . . . . . 535
Clark, George E., M. D., Hydrotherapy in Appendicitis . 282
Horace, M. D., Follicular Hypertrophy at the base of the Tongue . . . . . . 143
Club-Foot, new Features and Treatment of . . . . . . . . . . . . 705
Code of Ethics and the Cleveland Medical Society . . . . . . . . 560
Collyria, Abuse of . . . . . . . . 96
Trikresol as an Antiseptic for . . . . . . . . . 97
Conklin, W. L., M. D., Etiology, Diagnosis and Treatment of Diphtheria . . . . . . . . . . . . 640
Consumption, Recent Measures adopted by Department of Health toward Prevention of . . . . . . 746
Strychnine Treatment of . . . . . . 614
to Avoid and to Prevent . . 611

**Correspondence:**

Charcot, Monument Fund, James W. Putnam, M. D. . . 574
Hay, Mrs. Lyman T. . . . . . . . 361
Multiple Papillomes of the Larynx, Dr. A. Rosenberg, Berlin . . . . . . . . . . 165
Philadelphia Letters from J. P. . . . . . . . . . . . 111, 162

PAGE.
Cott, George F., M. D., Surgery of the Accessory Sinuses . . . . . . 321
Curtis, Harlow C., A. M , Address to Graduates, Niagara University 1
Cyst, Parovarian Complicating Pregnancy . . . . . . . . . . . . . . 281

DANIELS, CLAYTON M., M. D., two Peculiar Cases of Hernia . . . . . . . . . . . . 395
Dermatological Cases in Kaposi's Clinic . . . . . . . . . . . . . . . 136
Diphtheria . . . . . . . . . . . . . 596
Antitoxin Treatment of . . 172
Antitoxin in . . . . . . . 656
Etiology, Diagnosis and Treatment of . . . . . . 641
How Is It Propagated? . . 9
Points in the Etiology, Pathology and Treatment of . . . . . . . . . . . 288
Treatment of with Antitoxin . . . . . . . . . 400
Turpentine in . . . . . . 294
Dorr, Samuel G., M. D., Antitoxin in Malignant Diphtheria . . . 473
Doyle, Dr. A. Conan, an American, Retrospect by . . . . . . . . . 363
Doyle, Gregory, M. D., New Features in the Treatment of Club-Foot . . . . . . . . . . . . . . . 705
Dress Reform Agitation . . . . . . 619
Drifting,—Who, How, Whither? . 754
Dyspepsia, Atonic . . . . . . . . 334

EAR, Proliferous Inflammation of the Middle . . . . . . . 65
Elsner, Henry L., M. D., Clinical and Bacteriological Aspect of Mixed Infection in Pulmonary Tuberculosis . . . . . . . 204
S. L., M. D., Observations on an Anencephalic Monster . . . . . . . . . . 462
Enteroclysis in Enterocolitis and Zymotics . . . . . . . . . . . . . 663
Epileptics, Craig Colony for . . . . 556
Provision for . . . . . 18
Study of Reflexes in . . . 105
Epilepsy, Jacksonian, due to Gastric Auto-intoxication . . . . . . 104
Examinations, Analysis of Results of State Licensing . . 541
Regents, Fraud at . . . . 557
Examiners, Annual Report of State Board of Medical . . . . . . . . 488

PAGE.
Eye, Affections of, Dependent on Scrofulous Diathesis . . 76
an Ancient Treatise on Diseases of . . . . . . . 307

FAITH CURE, Reverse to the So-called . . . . . . . . . . 620
Feather Beds, Infected . . . . . . 613
Fee Bill, Old-fashioned County Medical . . . . . . . . . . . . . . 39
Filters, Application of Intermittent Filtration to Domestic . . . . . . 449
Fog Horn Nuisance . . . . . . . . 689
Ford, Willis E., M. D., Dangers of Uterine Tampon . . . . . . . . . 588
Four Years' College Course . . . . 688
Fractures of the Lower Extremities, Ambulatory Treatment in . . . . 221
Fresh Air Mission Hospital, Buffalo 35
Fund, the Mr. Tait . . . . . . . . 64

GANGRENE, Hospital, of Diphtheritic Origin . . . . . . 592
Germ Theory of Disease, a Criticism of . . . . . . . . . . . . 257
Glasses, Note on Smoked . . . . . 666
Glycerin, Dangers of Injection of, to Induce Premature Labor . . . 172
Gold Preparations in Skin Diseases and Syphilis . . . . . . . . . . 546
Goler, G. W., M. D., Diphtheria: How Is It Propagated? . . . . . 9
Gonorrhea, New Observations in . . 551
Grosvenor, J. W., M. D., Hot Springs, S. D. . . . . . . . . . 513

HALL, A. L., M. D., the Home Treatment of Hay Fever . . 328
Hartwig, M., M. D., Ambulatory Treatment in Fractures of the Lower Extremities . . . . . . . 221
Hay Fever, the Home Treatment of 328
Health Department, Statistics of . . 425
Resort, Cape May as a . . 750
Hernia, Causes of with a New Theory . . . . . . . . 129
Complicated . . . . . . . . 607
two Peculiar Cases of . . 395
Herniotomy, Double Femoral . . . 301
Hinkel, F. W., M. D., Review of the Relations Between the State and the Medical Profession . . . 193
Hip Disease . . . . . . . . . . . 433
Hospital, Munificent Gift to Buffalo General . . . . . . . . . . . 554
Hot Springs, S. D., a Health Resort . . . . . . . . . . . . . . . . 513

PAGE.
Hot Springs Meeting, Address of Hon. W. H. Martin at . 361
Echoes of . . . . . . . . . 361
Railroad Facilities to . . . 362
Howe, Lucien, M. D , Proliferous Inflammation of the Middle Ear . 65

### Hospital Notes:

Buffalo Fresh Air Mission Hospital . . . . . . . . . . . . 759
Buffalo General Hospital . 177, 759
Buffalo Hospital of the Sisters of Charity . . . . . . . . . . 759
Buffalo State Hospital . . . . 119
Erie County Hospital Examination . . . . . 694
Hospital of the Medical College of Virginia . . . . . . . . . . 176
Masonic Hospital, Chicago . . 432
Samaritan Hospital for Women, Montreal . . . . . . . . . . . 622
Stormont, Jane C , Hospital and Training School . . . . . . 382

Hubbell, Alvin A., M. D., Atrophy of Optic Nerve in Diseases of the Nervous System . . . . . . . . . . 531
Eye Affection Dependent upon Scrofulous Diathesis . . . . . . . . . 76
Hydrocele, New Treatment for . 587
Hygiene, Report of Committee on, to Investigate Water, Milk and Ice Supply . . . . . . . 714
Hysteria, Case with a Temperature of 105.8° . . . . . . . . . . . 300

IDIOCY . . . . . . . . . . . . . 344
Industrial School, Rochester State . . . . . . . . . . . . 36
Insane, Specific Gravity of the Urine of the . . . . . . . 105
Hospital Relations of the . 525
Insomnia, Treatment of . . . . . 367

### Illustrations:

Abdominal Pregnancy (Williams) . . . . . . . . . . . . 393
Ambulance Car (St. Louis Health Department) . . . . 497
Ambulatory Splint (Hartwig) . 222
An Anencephalic Monster (Angell-Elsner) . . . . . . . . 463
Antitoxin in Buffalo, Bacteriologists Inoculating a Horse 421
Buffalo General Hospital . . . 555

PAGE.
Club-Foot (Doyle). Fig 1. Patient Walking, showing feet everted by Spiral Rotator . . 707
Fig. 2. Patient Sitting, showing Flexibility of Spiral Rotator . . . . . . . . . . . . 707
Helmholtz, Professor . . . . . 251
Holmes, Oliver Wendell, Facing . . . . . . . . . . . . . . 249
King Thermometer Syringe . . 752
Nasal Stenosis (Mitchell) . . . 580
Neurological Bust (Krauss) . . 471
Neurologists' Percussion Hammer (Krauss) . . . . . . . . 416
School Wardrobe . . . . . . . 559
Statue of J. Marion Sims, Facing . . . . . . . . . . . . . . 303

### Instruments, New:

King Thermometer Syringe . . 752

KANSAS Medical Practice Bill, Defeat of . . . . . . . . . 553
Kidneys, Effect of Mercury on . . . 91
Kindergarten Association, Buffalo Free . . . . . . . . . . . . . . . 367
Kirk, William Redin, M. D., Constitutional Treatment of Diseases of the Female Sexual Organs . . 341
Krauss, Wm. C., M. D., a Neurological Bust . . . . . . . . . 470
Neurologists' Percussion Hammer . . . . . . . . . 416
Treatment of Neurasthenia 132

LABOR, Conduct of, through External Examinations. . . . . 108
Lanphear, Emory, M. D., Diphtheritic Hospital Gangrene—Relation of Tubal Pregnancy to Pelvic Hematocele—Peritoneal Adhesions . . . . . . . . . . . . 592
Laxol . . . . . . . . . . . . . . 62

### Leading Articles:

American Association of Obstetricians and Gynecologists . . . . . . . . . . . . . 166
Antitoxin in Buffalo . . . . . . 420
Buffalo University Medical College . . . . . . . . . . . 678
Care of the Poor . . . . . . . . 360
College Commencements and Alumni Association Meetings 678
Helmholtz, Professor . . . . . 251
Holmes, Oliver Wendell . . . 249

PAGE.

Mississippi Valley Medical Association . . . . . . . . . . 303
National Public Health Establishment . . . . . . . . . . 359
Niagara University Medical College . . . . . . . . . 682
Our Semi-centennial Anniversary . . . . . . . . . . . . 753
Pelvic Inflammatory Disease—What Is True Conservatism in Its Treatment? . . . 552
State Examination for Medical License . . . . . . . . . . 492
State Licensure for the Practice of Medicine . . . . . . 33
Summer Intestinal Diseases of Children . . . . . . . . . . 114
Vaginal Douching During the Puerperium . . . . . . . . 615

Lenses, Therapeutic Value of Weak 100
Letchworth, Wm. Pryor, L.L. D., Provision for Epileptics . . . . . 18
Licenses from other State Examining Boards, Indorsements of . . . 609
Lister, Sir Joseph, Testimonial to . 364

**Literary Notes:**

Albinism, Collective Investigation of . . . . . . . . 445
Alvarenga Prize . . . . . . 320
American Journal of Insanity . 60
American Lancet . . . . . . . 574
A New System of Surgery . . . 186
An Intramural View . . . . . 186
Annales d'Oculistique . . . . . 576
Annales de l'Institut Ste. Anne 639
Atlantic Medical Weekly . . . 445
Author of Gallegher . . . . . . 61
Bacteriological Laboratory, Parke, Davis & Co. . . . . 511
Blakiston, P. Son & Co., Calendar . . . . . . . . . . . . 383
Bulletins of the Harvard Medical Alumni Association . . . 382
Calendar, McArthur Hypophosphite Co.'s . . . . . . . . 512
Columbus Medical Journal . . 61
Columbia Calendar . . . 384
Davidson Rubber Co., Calendar . . . . . . . . . . . . 384
Doctor and Druggist . . . . . 60
F. A. Davis Company . . . . . 61
Germania . . . . . . . . . . 639
Gould's New Medical Dictionary . . . . . . . . . . . 447
Health, Sanitation and Climatology . . . . . . . . . . . . 768
Hot Springs Medical Journal . 62

PAGE.

Illustrated Surgical Clinic, by Bernays . . . . . . . . . . . 640
Index Medicus . . . . . . . . . 384
International Medical Annual . 446
Johnson & Johnson . . . . . . . 445
Journal of Medicine and Science . . . . . . . . . . . 384
Journal of the American Public Health Association . . . . . . 445
Journal of Practical Medicine . 639
Kola, Another Book on . . . 704
Ladies' Home Journal . . . . . . . . . . 128, 446, 638, 704
Lehigh Valley Medical Magazine . . . . . . . . . 60
Lindsay & Blakiston's Visiting List . . . . . . . . . . . . . 319
Mathews's Medical Quarterly . 371
McArthur's Vest Pocket Diary 383
Medical and Surgical Reporter 61
Medical Chronicle . . . . . . 704
Medical Fortnightly . . . . . 62
Medical Mirror . . . . . . . . 320
Medical Novelty Co.'s Pocket Daybook and Visiting List . . 576
Medicine . . . . . . . . . . . 638
Monatsschrift für Geburtshülfe und Gynäkologie . . . . . . . 512
New Medical Journals . . . . . 120
New York Pharmaceutical Co. 383
North Carolina Medical Journal . . . . . . . . . . . . 382
Obstetric Surgery, Grandin and Jarman . . . . . . . . . . . 127
Odontographic Journal . . . . 502
Oldest Medical Journals . . . . 575
Physicians and Surgeons of America . . . . . . . . . . . 127
Physician's Pocket Diary, Mellier Drug Co. . . . . . . . . . 512
Richmond Journal of Practice . 127
Rio Chemical Co.'s Map of the United States . . . . . . . . 639
Schulze-Berge & Koechl, Advertisement of . . . . . 383, 445
Scientific Bibliography, Bailliere & Son . . . . . . . . . 511
Southern California Practitioner . . . . . . . . . . . 576
St. Louis Clinique . . . . . . 60
Surgery 200 Years Ago . . . . 512
Suggestive Therapeutics in Psychopathia Sexualis . . . 446
Temperature Chart, Laine's . . 383
Twentieth Century Practice . . 447
Warner's Therapeutic and Ready Reference Book . . . 576
William R. Warner & Co.'s Decoration . . . . . . . . . 703
Woman's Medical Journal . . . 60

PAGE.

Woman's Medical Journal Calendar . . . . . . . . . . . . 447
Youth's Companion . . . . . . 319

Litter, Committee on Military . . . . 491
Lunacy, State Commissioners in . . 666

MARASMUS . . . . . . . . . . 15
Marcus, Herman D., M. D., Marasmus . . . . . . . . . . . . . 15
Mann, Frederick J., M. D., Vesicle Calculi in the Female . . . . . . 536
Maryland Medical Journal on State Examinations . . . . . . . . . 619

**Medical College Notes:**

Albany Medical College . . . . 694
Birmingham Medical College . 44
Buffalo University . . . . . . . . . . . 122, 248, 432, 627
Cincinnati College of Medicine and Surgery . . . . . . . . 248
College of Physicians and Surgeons, St. Louis . . . . . 121
Department of Pedagogy, University of Buffalo . . . . . . 694
Jefferson Medical College . . . 759
Kansas City Medical College . 45
Medical Department of the University of Wooster . . . 45
New York Medical College, Sketch of . . . . . . . . . 366
Niagara University . . . . 122, 626
University of Michigan . . . . . 502
University of Pennsylvania . . 44

Medical Corps U. S Navy . . . . . 686
Medical Education, Opposition to Improved Methods of . . . . . 556
Medical Practice Act in Georgia . . 365
Medical Practice Law, Amendments to . . . . . . . . . . 675
Medical Profession in New York, Relations between the State and . 193
Medical Society of State of New York vs. New York State Medical Association . . . . . . . 554
Medicine, Restriction by the State of the Right to Practise . . . . . 308
Memorial Exercises of Dr. A. L. Loomis . . . . . . . . . . . . . 686
Meningitis due to Typhoid Bacillus 287
Menstruation, Note on the Processes of . . . . . . . . . . . . 385
Metric Prescriptions . . . . . . . 160
Midwives, Ignorance of . . . . . . 171
Milk Supply of Buffalo . . . . . . 668

PAGE.

**Miscellany:**

Anatomical Material, Collection and Preservation of . . 186
Carpenter Milk Company . . . 189
Charles Roome Parmele Co. . 191
Civil Service Examination for Physicians in State Hospitals 318
Crystal Water Company . . . 768
Danger in Colored Paper . . . 191
Doliber-Goodale Company . . 768
Influence of Climate on Health 448
Legality of Vaccination . . . . 192
Lindsay & Blakiston's Visiting List . . . . . . . . . . . . 319
Medical Examinations in New York . . . . . . . . . . . 640
North Carolina Board of Medical Examiners . . . . . . . 192
New York Physicians' Mutual Aid Association . . . . . . . 640
Numerical Strength of Different Schools of Medicine in the United States . . . . . . 89
Pension for Billroth's Widow . 191
Phrenology a Humbug . . . . 188
Partnership . . . . . . . . . . 640
St. Louis Electrical Ambulance Car . . . . . . . . . . . . 192
Virchow's Day's Work . . . . 191
When in Doubt, Operate . . . 190

Mississippi Valley Medical Association, Action on Ethics by 362
Pleasant Episodes at . . . 363
Mitchell, S., M. D., Nasal Stenosis 577
Monster, Observations on an Anencephalic . . . . . . . . . . . . 462
Montana, Medical Legislation in . 667
Mynter, Herman, M. D., Nephrectomy for Nephrolithiasis and Pyonephrosis . . . . . . . . . . 388

NASAL STENOSIS . . . . . . 577
Nature, Freak of . . . . . . 618
Nephrectomy for Nephrolithiasis and Pyonephrosis . . . . . . . . 388
Neurasthenia, the Treatment of . . 132
Newspaper Fraternity *in re* Advertising Physicians . . . . . . . . 36
New York Central and Hudson River Railroad . . . . . . . . . 384
Niagara University Medical College, Address to Graduates of . . 1
Niagara Water . . . . . . . . . . 603

OFFICE HOURS, Abolishment of Evening . . . . . . . . . 365

PAGE.

**Obituary :**

Andrews, Judson Boardman . . . . 93
Briggs, William Thompson . . 41
Bull, Louis Alexander . . . . 369
Byron, John W. . . . . . . . . 691
Dabney, William Cecil . . . . 175
Dunnigan, Joseph H. . . . . . 429
Dujardin-Beaumetz, G. S. . . 564
Goldthwaite, Henry . . . . . 430
Loomis, Alfred Lebbeus . . . 431
Miles, Albert B. . . . . . . . 174
Miner, David Worthington . . 431
Nichols, James . . . . . . . . 501
Rowland, M. C. . . . . . . . . 429
Sim, F. L. . . . . . . . . . . . 370
Tuke, Daniel Hack . . . . . . 464

Omentum, the Lesser . . . . . . . 520
Ophthalmia Neonatorum, Blindness from . . . . . . . 98
Purulent, Treatment of . . 99
Ophthalmological Congress, Dr. Hubbell's Report of . . . . 366, 562
Optic Nerve, Atrophy of in Nervous Diseases . . . . . . . . . 531
Oxytocic, Salicylic Acid as an . . . 475

PARAGRAPHS Borrowed without Credit . . . . . . . . . 557
Park, Roswell, M. D., Sarcoma of Tonsil—Cancer of Breast . . . . 397
Pelvic Cellulitis, Cases of True—a Plea for more thorough Pelvic Surgery . . . . . . . . . . . . 710
Pension Fund for Health Department Employees . . . . . . . . 119
Percussion Hammer, a Neurologist's . . . . . . . . . . . . . 416

**Personals :**

Bacon, John E. . . . . . . . . 756
Bartow, Bernard . . . . . . . 563
Benedict, A. L. . . . . . . 428, 500
Bissell, William G . . . . . . 428
Blaauw, E. E. . . . . . . . . . 500
Bryant, Joseph D. . . . . . . 428
Bryant, Percy . . . . . . . . . 427
Burnett, Swan M. . . . . . . . 120
Burr, C. B. . . . . . . . . . . 121
Buswell, Henry C. . . . . . . 427
Carpenter, Thomas B. 173, 428, 756
Carroll, Evangeline . . . . . . 40
Carroll, Jane W. . . . . . . . 40
Chaddock, Charles Gilbert . . 310
Clark, Edward . . . . . . . . . 563
Clark, Horace . . . . . . 39, 755
Congdon, Charles E. . . . . . 621
Cordier, A. H. . . . . . . . . 428

PAGE.

Cushing, Clinton . . . . . . . 369
Davis, John D. S. . . . . . . . 499
Davis, William E. B. . . . . . 499
Diehl, Alfred E. . . . . . . . . 254
Fritz, William C. . . . . . . . 756
Gibney, Virgil P. . . . . . 39, 432
Gilray, E. J. . . . . . . . . . . 369
Grant, Henry Y. . . . 369
Greene, Walter D. . . . . . . . 756
Hamilton, Allen McLane . . . 692
Harper, J. G. . . . . . . . . . 254
Hinkel, F. W. . . . . . . 192, 621
Holmes, J. B. S. . . . . . . . . 40
Hopkins, H. G. . . . . . . . . 621
Hubbell, A. A. . . . . . . . 40, 174
Hurd, Arthur W. . . . . . . . 309
King, Willis P. . . . . . . . . . 369
Knight, Charles Huntoon . . . 120
Krauss, William C. . . . 120, 174
Krug, J. F. . . . . . . . . . . . 174
Lewis, Daniel . . . . . . . 428, 500
Lothrop, Thomas . . . . . . . . 120
Lydston, G. Frank . . . . 39, 192
Mann, M. D. . . . . . . . . . 40
Mathews, Joseph M. . . . . . 310
McMurtry, L. S. . . . . . . . 428
Moehlau, F. G. . . . . . . . . 120
Mulford, Henry J. . . . . . . . 692
Murphy, J. B. . . . . . 369, 755
Mynter, Herman . . . . . . . . 756
Osthues, Henry . . . . . . . . 120
Park, Roswell . . . . . . . . . 500
Parmenter, John . . . . . . . 621
Pattison, George W. . . . . . 692
Pohlman, Julius . . . . . 174, 563
Pond, Edmund M. . . . . . . . 368
Praeger, E. Arnold . . . . . . 121
Quirk, H. W. . . . . . . . . . 563
Reed, Charles A. L. . . . . . 563
Reynolds, C. J. . . . . . . . . 40
Ring, Charles A. . . . . . . . 174
Ring, William G. . . . . . . . 621
Roberts, John B. . . . . . . . 40
Rogers, H. W. . . . . . . . . . 500
Rolph, R. T. . . . . . . . . . . 500
Ross, Frank W. . . . . . . . . 310
Satterlee, Richard Henry . . . 120
Sexton, John C. . . . . . . . . 254
Snow, Irving M. . . . . . . . . 756
Spratling, William P. . . . . 368
Stanton, Byron . . . . . . . . 121
Stone, I. S. . . . . . . . . 40, 254
Thorn, S. S. . . . . . . . . . . 39
Thornbury, Frank J. . . . . . 692
Trowbridge, G. R. . . . . . . 428
Vander Veer, Albert . . . . . 499
Wetmore, S. W . . . . . . . . 621
Wetmore, Mary B. . . . . . . 621
Williams, Herbert U. . . . . . 310
Williams, Sir John . . . . . . 40

PAGE.
Wishard, W. N. . . . . . . . . 563
Wright, Adam H. . . . . . . . 499

Peritoneal Adhesions . . . . . . . 592
Philadelphia Notes . . . . . . . . 30
Phthisis, Creosote for . . . . . . 113
Decision Relating to, in Toronto . . . . . . . . 17
Pork, New Microörganism Discovered in . . . . . . . . . . 608
Positions, Occipito–Posterior . . . 466
Postmaster-General, Report of . . 368
Pregnancy, Abdominal, Complicated with Pyosalpinx . . . 392
Kidney of . . . . . . . . . 358
Tubal, Relation of to Pelvic Hematocele . . . . . 592
Prize, the Alvarenga . . . . . . . 320
Pronunciation, Medical . . . . . . 426
Putnam, James W., M. D., Idiocy . 344
Pyrexia, in Acute Diseases of Childhood . . . . . . . . . . . . . 604

**Progress in Medical Science:**

Neurology, by William C. Krauss, M. D. . . . . . . . 104
Ophthalmology, by Alvin A. Hubbell, M. D. . . . . . . 96
Pediatrics, by Maud J. Frye, M. D. . . . . . . . . . . . 604
State Medical Examinations, by William Warren Potter, M. D. . . . . . . . . . . 609, 675
State Medicine, Public Health, Hygiene and Bacteriology, by Ernest Wende, M. D., 596, 668, 746

QUACKS in Buffalo . . . . . . . 115

RAFTER, GEORGE W., Application of Intermittent Filtration to Domestic Filters . . . 449
Rauch, Dr. John H., and the Massachusetts Medical Society . . . . 117
Rebuke, Deserved, to the Chicago Summer School of Medicine . . . . . . . . . . 495
a Just, to New York Life Insurance Co. . . . . . . 558
Rectal Tube, Dangers of the Long . 32
Ring, Charles A., M. D., Our Insane Patients and Their Hospital Relations . . . . . . . . . . . 525
Robinson, Byron, M. D., Causes of Hernia, with a New Theory . . . . . . . . 129
The Lesser Omentum . . . 520

PAGE.
Rush Monument, Report on, by Dr. Gihon . . . . . . . . . . . . . 687

**Reviews:**

Abbott: Principles of Bacteriology . . . . . . . . . . . . 380
Abrams: Clinical Diagnosis . 58
Abrams: Transactions of the Antiseptic Club . . . . . . 766
Adams: Life of D. Hayes Agnew . . . . . . . . . 569
Adams: Diagnosis and Treatment of Diseases of the Eye . 572
Annual Report of the State Board of Health, Illinois . . 376
Apostoli: Travaux d' Electrotherapie Gynecologique . . . 437
Attfield: Medical and Pharmaceutical Chemistry . . . . . 573
Bartley: Medical and Pharmaceutical Chemistry . . . . 572
Beard: Sexual Neurasthenia 700
Beck: Theory and Technique of Surgical Asepsis . . . . . 760
Biddle: Materia Medica and Therapeutics . . . . . . . . 699
Bigelow: International System of Electro-Therapeutics 46
Billings and Hurd: Suggestions to Hospital Visitors . . 702
Bissell: Manual of Hygiene . 442
Book of the New York State Reformatory . . . . . . . 439
Bourges: Treatise of Diphtheria . . . . . . . . . . . . . 181
Bread from Stones . . . . . . 636
Buchanan: Antiseptics and Antisepsis . . . . . . . . . 701
Buck: Diseases of the Ear . . 634
Cerna: Notes on Newer Remedies . . . . . . . . . . . . . 635
Cheyne: Treatment of Wounds, Ulcers and Abscesses . . . . 762
Corning: Headache and Neuralgia . . . . . . . . . . . 56
Corning: Pain . . . . . . . 56
Curtis: General Medicinal Technology . . . . . . . . . 764
Da Costa: Manual of Modern Surgery . . . . . . . . . . . 436
Daland: International Clinics, V. Series, Volume I. . . . . 763
Davis: Manual of Obstetrics 55
Dench: Diseases of the Ear . 568
Education, Report of thè Commissioner of . . . . . . . . 125
Finger: Gonorrhea . . . . . 54
Flint: Practice of Medicine . 374

PAGE.
Gould: Dictionary of Medicine . . . . . . . . . . . . . 51
Gleason: Essentials of Diseases of the Ear . . . . . . 378
Graphic History of the Fair . . 182
Grandin: Obstetric Surgery . 630
Guernsey: Urinalysis, including Blanks . . . . . . . . . 765
Hamilton: System of Legal Medicine . . . . . . . 313, 627
Hare: Practical Therapeutics 375
Herman: Difficult Labor . . . 631
Hughes: Practice of Medicine 633
Index Catalogue of Surgeon-General's Library . . . . . 181
Ingals: Diseases of the Chest, Throat and Nasal Cavities . 435
International Clinics, Fourth Series, Vol. I., 125; Vol. II., 378; Vol. III., 440; Vol. IV., 633
Jackson: Essentials of Diseases of the Eye . . . . . . 183
Johns Hopkins Hospital Reports, Typhoid Fever, 312; Surgery II, Vol. IV., 440; Neurology II. . . . . . . . . 570
Keating and Coe: Clinical Gynecology . . . . . . . . 695
Kimber: Anatomy and Physiology for Nurses . . . . . . 433
Krieger: Blood Serum Therapy and Antitoxins . . . . . 509
Lefart: Macadies du Cœur et de L'Appareil Circulatoire . 767
Leonard: Manual of Bandaging . . . . . . . . . . . . . 701
Leonard: Pocket Anatomist . 442
Linn: Where to Send Patients for Water Cure and Climate Treatment . . . . . . . . . 181
Long: Syllabus of Gynecology 573
MacFarlane: Clinical Manual . . . . . . . . . . . . . 183
Madden: Clinical Gynecology 124
Morris: Diseases of the Skin 179
Morten: Nurse's Dictionary 182
Manton: Human Embryology 379
Medical Register of New York, New Jersey and Connecticut . . . . . . . . . . . . 377
Morrow: System of Genito-Urinary Diseases . . . . . . 311
Nancrede: Essentials of Anatomy . . . . . . . . . . 126
Noyes: Diseases of the Eye . 53
Purdy: Urinalysis and Urinary Diagnosis . . . . . . 571
Quain: Dictionary of Medicine . . . . . . . . . . . . . 434
Raymond: Human Physiology 379

PAGE.
Reeves: Medical Microscopy 373
Regis: Manual of Mental Medcine . . . . . . . . . . . . . 509
Report of Commissioner of Education, 1892–1893 . . . 763
Report of the Michigan State Board of Health, 1892 . . 767
Report of St. Lawrence State Hospital, 1894 . . . . . . . 762
Report of State Board of Charities, 1893 . . . . . . . . . . 314
Report of State Board of Charities, 1894 . . . . . . . . . . 764
Report of State Board of Health 571
Robinson: Landmarks in Gynecology . . . . . . . . . . 438
Robinson: The Sympathetic Nerve . . . . . . . . . . . 381
Rohé: Text-Book of Hygiene 441
Roosa: Diseases of the Eye . 505
Sajous: Annual of the Universal Medical Sciences . . . 179
Sänger: Asepsis in der Gynäkologie und Geburtshülfe . 180
Sayre: Essentials of Pharmacy . . . . . . . . . . . . 58
Schrenck-Notzing: Suggestive Therapeutics in Psychopathia Sexualis . . . . . . . 760
Shaw. Essentials of Nervous Diseases and Insanity . . . . 126
Shoemaker: Materia Medica and Therapeutics, Vol. II. . 437
Skene: Medical Gynecology . 761
Stedman: Twentieth Century Practice, Vol. I., 629, Vol. II., 700
Steiner: History of Education in Connecticut . . . . . 566
Sternberg: Immunity, Protective Inoculations and Serum-Therapy . . . . . . . . . . . 765
Stevens: Practice of Medicine 438
Stewart: Practice of Medicine 443
Stoddart Brothers, Catalogue of Instruments and Drugs . . 635
Stone: Biography of American Physicians and Surgeons . . 50
Stuart: Treatment of Typhoid Fever . . . . . . . . . . . . . 181
Sutton: Tumors — Innocent and Malignant . . . . . . . 49
Taylor: Index of Medicine . . 766
Thomas: Diet Lists and Sickroom Dietary . . . . . . . . 766
Thornton: Manual of Prescription Writing . . . . . . 702
Tillmans: Principles of Surgery and Surgical Pathology 632
Transactions Association of Military Surgeons . . . 435

PAGE.

Transactions of the Association of American Physicians, 1894 . . . . . . . . . . 314
American Dermatological Association . . . . . . . 58
American Gynecological Society, Vol. XIX. . . . 314
American Laryngological Association . . . . . . 181
American Laryngological Association, 1894 . . . . 764
American Surgical Association . . . . . . . . . 380
Colorado State Medical Society . . . . . . . . 634
Indiana State Medical Society . . . . . . . . . . 180
Michigan State Medical Society . . . . . . . . 441
Medical and Chirurgical Faculty, State of Maryland . . . . . . . . . . 315
Medical Society of the State of New York . . . 375
Medical Society of the State of Pennsylvania . 433
Medical Society of the State of North Carolina . . . . . . . . . . 766
State Medical Association of Texas . . . . . . . . 376
Waite: Materia Medica, Pharmacology and Therapeutics . 377
Warren: Surgical Pathology and Therapeutics . . . . . . 698
Weekly Abstract of Sanitary Reports, Marine Hospital Service . . . . . . . . . . . 767
Wimmer: Physicians' Vade Mecum . . . . . . . . . . . 702
Witthaus and Becker: Medical Jurisprudence, etc. . . . 503
Witthaus: Essentials of Chemistry and Toxicology . . . . 443
Woodhead: Practical Pathology . . . . . . . . . . . . . 57
Worcester: Small Hospitals . 177
Year Book of Treatment for 1895 . . . . . . . . . . 636
Young: Orthopedic Surgery . 372

SANITARIA and Private Hospitals . . . . . . . . . . . . . 171
Saligenin in Articular Rheumatism. 751
Sarcoma of the Tongue Treated with Pyoktanin . . . . . . . . . 148
Satterlee, Richard H., M. D., Note on Smoked Glasses . . . . . . . 666

PAGE.

Schieffelin, W. H. & Co., 100 Years of Business Life . . . . . . . . 117
School Wardrobe, Sanitary . . . . 558
Sexual Organs, Constitutional Treatment of Diseases of the Female . 341
Sheppard Asylum, Report of . . . . 423
Sims, J. Marion, Bronze Statue of . 309
Sinuses, Surgery of the Accessory . 321
Sleeping Cars, Sterilization of . . . 116

**Society Meetings:**

American Academy of Medicine 501
American Academy of Railway Surgeons . . . . . . . . . . 255
American Association for the Advancement of Science . . . 44
American Association of Obstetricians and Gynecologists . . . . . . . . 41, 122, 758
American Electro-Therapeutic Association . . . . . . 43, 758
American Medical Association 624
American Medical Publishers' Association 43, 175, 566, 624, 756
American Microscopical Society . . . . . . . . . . . . . 44
American Public Health Association . . . . . . . . . . . . 43
Association of American Medical Editors . . . . . . . . . 123
Association of Erie Railway Surgeons . . . . . . . . . . . 370
Association of Military Surgeons of the United States . . . . . . . . . . 564, 624, 693
Buffalo Academy of Medicine . 176
International Congress of Dermatology . . . . . . . . . . . 622
Lehigh Valley Medical Association . . . . . . . . . . . . 44
Medical Association of Central New York . . . . . 44, 123, 254
Medico-Legal Congress . . . . 625
Medical Society of the County of Erie . . . . . . . . 370, 694
Medical Society of the State of New York . . . . . . . . . 371
Medical Society of Virginia . . 176
Mississippi Valley Medical Association . . . . . . . . 176, 757
Mitchell District Medical Society . . . . . . . . . . . . . 757
National Confederation of State Medical Examining and Licensing Boards . . . . . . 757
New York State Association of Railway Surgeons . . . . 176
Ohio State Medical Society . . 624

PAGE.

Southern Surgical and Gynecological Association . . 175, 371
Tri-State Medical Society of Alabama, Georgia and Tennessee . . . . . . . . . . . . 179
Tri-State Medical Society of Iowa, Illinois and Missouri . 565

**Society Proceedings:**

American Association of Obstetricians and Gynecologists . . . . . . . . . . . . 223
Buffalo Academy of Medicine . . . . . . . . . . 247, 714
Buffalo Academy of Medicine, Surgical Section . . . . . . 356
Louisville Surgical Society . . 85
Medical Association of Central New York . . . . . . . . . . 242
Medical Society of the County of Chautauqua . . . . . . . 31
Medical Society of the County of Erie . . . . . . 92, 412, 745
Ophthalmological Congress, Eighth International . . . . . . . . . 295, 346, 402, 476, 537
Sponges from Absorbent Cotton, etc. . . . . . . . . . . . . . . . . 31
Stafford, James, M. D., Hysteria with a Temperature of 105.8° . . 300
State Examinations for Medical License . . . . . . . . . 170
New York . . . . . . . 253, 306
Pennsylvania . . . . . . . 253
State Board of Medical Examiners, Report of . . . . . . . . . 488
Stearns, Frederick P. & Co. . . . . 62
Strike, the Great Railroad, at Chicago . . . . . . . . . . . . . . 38
Suppuration, Tincture of Iodine in 524
Syphilitic Infection, Extra Genital 109
Symphysiotomy, Revival of . . . . 152
Syringe, King Thermometer . . . . 752

TAIT, Mr. LAWSON, Correction by . . . . . . . . . . . 363
Criticism of the Germ Theory of Disease, Based on the Baconian Method 257
Note on the Processes of Menstruation . . . . . 385
Tax, an Unjust . . . . . . . . . . 689
Thanks, the Journal's Appreciative 617
Therapeutic Notes . . . . . . 160, 302
Thermometers, Certified Clinical . 248
Thornbury, Frank J., M. D., Kaposi's Dermatological Clinic . . . . . . . . . . 136

PAGE.

Thornbury, Frank J., M. D., New Microörganism in Pork . 608
The Pathology of Trichinosis . . . . . . . . . . 332
Trichinosis, the Pathology of . . . 332
Trional in Insomnia of Children . . 667
Tonsil, Sarcoma of . . . . . . . . 397
Tonsilitis, Salicylate of Sodium in 147
Tongue, Sarcoma of, Treated with Pyoktanin . . . . . . 148
Hypertrophy of Follicular Glands at Base of . . . . 143
Topics of the month 35, 115, 168, 307, 363, 423, 495, 554, 617, 686, 754
Trowbridge, G. R., M. D., State Commissioners in Lunacy . . . . 666
Tuberculosis, Clinical and Bacteriological Aspect of Mixed Infection in . . . 204
Mortality of . . . . . . . . 63
Rules for Prevention of . . 418
Tuberculous Sputum . . . . . . 603
Typhoid Fever, Easy Method of Bathing in . . . . . . . 106
Investigations of Outbreak in 1893 . . . . . . . . 718

UNIVERSITY of the State of New York . . . . . . . . . 168
Unkind Words in Medical Journals 119
Urethritis, Simple and Specific . . . 85
Uterine Tampon, Dangers of . . . 588
Urine, Easy and Delicate Tests for Albumin in . . . . . . . . . . 551

VAGINAL Injections After Labor . . . . . . . . . . . . 203
Van de Warker, Ely, M. D. Cases of True Pelvic Cellulitis, a Plea for more thorough Pelvic Surgery . . . . . . . . . . . . . . 710
Van Peyma, P. W., M. D., Occipito-Posterior Positions . . . . . 466
Verses Dedicated to the City Bug-Hunter . . . . . . . . . 690
Visiting List, the Medical News . . 382

WATER, Boiled, for School Children . . . . . . . . 302
Williams, H. T., M. D , Abdominal Pregnancy with Pyosalpinx, Laparatomy, Recovery . . . . . . . 392

YALE MEDICAL SCHOOL, Four Years' Course . . . . . 498
Young, A. A., M. D., Points in the Etiology, Pathology, and Indicated Treatment of Diphtheria 288

